# VITAMIN E IN HUMAN MILK AND ITS RELATION TO THE NUTRITIONAL REQUIREMENT OF THE TERM NEWBORN

**DOI:** 10.1590/1984-0462/;2017;35;2;00015

**Published:** 2017

**Authors:** Anna Larissa Cortês da Silva, Karla Danielly da Silva Ribeiro, Larisse Rayanne Miranda de Melo, Dalila Fernandes Bezerra, Jaluza Luana Carvalho de Queiroz, Mayara Santa Rosa Lima, Jeane Franco Pires, Danielle Soares Bezerra, Mônica Maria Osório, Roberto Dimenstein

**Affiliations:** a Universidade Federal do Rio Grande do Norte (UFRN), Natal, RN, Brasil.; b UFRN, Santa Cruz, RN, Brasil.; c Universidade Federal de Pernambuco (UFPE), Recife, PE, Brasil.

**Keywords:** Alpha-tocopherol, breast milk, lactation, recommended dietary allowances, infant

## Abstract

**Objectives:**

To determine the alpha-tocopherol concentration in breast milk at different periods of lactation and to estimate the possible supply of vitamin E to the infant.

**Methods:**

A longitudinal observational study was carried out with 100 mothers at University Hospital Ana Bezerra (HUAB), at Universidade Federal do Rio Grande do Norte, in Santa Cruz (RN), Northeast Brazil. Samples of colostrum (n=100), transitional milk (n=77), and mature milk (n=63) were collected. Alpha-tocopherol was analyzed by high-performance liquid chromatography. Vitamin supply to the newborn was estimated by comparing the nutritional requirement of vitamin E (4 mg/day) with the potential daily intake of milk.

**Results:**

The mean alpha-tocopherol concentration found in colostrum, transitional, and mature milk was 40.5±15.0 µmol/L, 13.9±5.2 µmol/L, and 8.0±3.8 µmol/L, respectively (p<0.001). The possible effect of these milks offered to the infant 6.2 mg/day of vitamin E in colostrum, 4.7 mg/day in transitional milk, and 2.7 mg/day in mature milk (p<0.0001), shows that only the mature milk did not guarantee the recommended quantity of this vitamin.

**Conclusions:**

Alpha-tocopherol levels in human milk decrease through the progression of lactation, and the possible intake of colostrum and transitional milk met the nutritional requirement of the infant. Mature milk may provide smaller amounts of vitamin E. Thus, it is important to study the factors that are associated with such low levels.

## RESUMO

**Objetivos:**

Determinar a concentração de alfa-tocoferol no leite materno em diferentes períodos de lactação e estimar o provável fornecimento de vitamina E ao lactente.

**Métodos:**

Estudo longitudinal observacional realizado com 100 puérperas atendidas para o parto no Hospital Universitário Ana Bezerra (HUAB) da Universidade Federal do Rio Grande do Norte (UFRN), em Santa Cruz (RN). Foram coletados leite colostro (n=100), leite de transição (n=77) e leite maduro (n=63) no seguimento da lactação. O alfa-tocoferol foi analisado por cromatografia líquida de alta eficiência. O fornecimento de vitamina E para o neonato foi estimado comparando-se o requerimento nutricional de vitamina E (4 mg/dia) com a ingestão diária de leite.

**Resultados:**

A concentração média de alfa-tocoferol encontrada nos leites colostro, de transição e maduro foi 40,5±15,0 µmol/L, 13,9±5,2 µmol/L e 8,0±3,8 µmol/L, respectivamente (p<0,001). A possível ingestão desses leites pelo lactente forneceu 6,2 mg/dia de vitamina E no colostro, 4,7 mg/dia no de transição e 2,7 mg/dia no maduro (p<0,0001), evidenciando que apenas o último não garantiu a quantidade recomendada dessa vitamina.

**Conclusões:**

Os níveis de alfa-tocoferol no leite diminuíram com a progressão da lactação, e a provável ingestão dos leites colostro e de transição conseguiu atender ao requerimento nutricional do lactente. O leite maduro pode fornecer menores quantidades da vitamina E, o que torna importante o estudo dos fatores que se associam a esses baixos níveis.

## INTRODUCTION

Vitamin E, a nomenclature used to describe compounds with biological activity of alpha-tocopherol, is a micronutrient liposoluble of utmost importance to the early stages of life, because it acts in defense against oxygen toxicity in the extrauterine environment and offers limited placental transfer to the fetus during the gestational period. Thus, maternal milk is responsible for supplying the demand of this vitamin to the neonate in this initial period and during lactation^1^ ^,^ ^2^ thereby protecting it from the development of signs and symptoms related to their disability, such as hemolytic anemia, bronchopulmonary dysplasia, neurological dysfunction, and increased neonatal mortality.^3^

The human milk provides nutritional, immunological, and emotional benefit to the infant’s health, as mothers are advocated to exclusively breastfeed their babies until they are six months of life. Its composition changes during the lactation period, in accordance with the needs of the newborn.^4^ It is known that the content of vitamin E decreases as the time of lactation increases, and that colostrum has the highest content of this vitamin.^5^

Some studies have determined the levels of alpha-tocopherol in breast milk while analyzing only one or two moments of lactation.^6^ ^,^ ^7^ However, when it comes to longitudinal studies, few consider the analysis of the vitamin in the three stages of lactation-colostrum, transitional, and mature,^4^ ^,^ ^8^ ^,^ ^9^, and this approach to estimate the supply of vitamin E for the infant is rarely done.

The *Dietary Reference Intake* (DRI) recommends the intake of 4 mg/day of vitamin E (alpha-tocopherol) for children between 0 and 6 months of life. This recommendation is based on a value of adequate intake (AI), determined by calculating the mean concentration of vitamin E in human milk produced during the first six months of lactation, and the average consumption of 780 mL of milk per day.^10^

Considering the importance of a nutrition which is adequate in vitamin E in the processes of disease prevention and health promotion in newborns, and the scarcity of longitudinal studies with the approach in the application of the infant, this work intended to evaluate the concentration of vitamin E (alpha-tocopherol) in three different stages of lactation - in colostrum (until the third day postpartum), in the milk of transition (between the 7^th^ and 15^th^ days), and in mature milk (30 days post-partum) - and to estimate the probable supply of vitamin from the intake of milk of nursing mothers.

## METHOD

This study is a longitudinal observational study type, composed of 100 pregnant women assisted at the University Hospital Ana Bezerra (HUAB), at the *Universidade Federal do Rio Grande do Norte* (UFRN), in Santa Cruz (RN), Brazil, during the period 2012 to 2014. The women who were taken into consideration were older than 18 years, without chronic diseases, who had been diagnosed and who had a vaginal delivery term (>37 weeks of pregnancy), in single birth, and without congenital malformation or syndromes. Those who received vitamin E supplementation during pregnancy and the postpartum period were excluded, in order to analyze the natural conditions of supply of vitamin to newborns.

After the explanations about the research, all participants signed the informed consent form (ICF), approved by the Ethics Committee of UFRN (CAAE 07416912.8.0000.5537). The general characteristics were obtained from the medical records of each patient.

There were three samples of milk from the same woman. The procedures were carried out in the outpatient clinic of the hospital or in home visit previously scheduled with the participant. All milk colostrum was collected at the hospital until three days after birth (n=100); the milk of transition, between 7 and 15 days after the birth (n=77), and the mature milk, around 30-40 days after the birth (n=63). The losses in the follow-up of the research were due to the interruption of breastfeeding, the changes in telephone contact or address without notice, which prevented the scheduling of the home visit, or the withdrawal of a participant.

The samples were obtained by manual expression of breast only, at the beginning and at the end of the feed, after an overnight fast from 8-12 hours. For the colostrum, there were two collections, the second being 24 hours after the first to correct possible variations in the concentration of alpha-tocopherol in the first secretions of milk.

The samples were collected in polypropylene tubes protected from light to prevent a possible vitamin loss, transported under refrigeration to the Laboratório de Bioquímica dos Alimentos e da Nutrição (LABAN), and stored at -20ºC in a nitrogen atmosphere for subsequent extraction and lipid analysis of alpha-tocopherol.

The analysis of alpha-tocopherol in milk was performed according to the adapted method of Ortega et al. ^11^ In 1 mL of milk, the same quantity of ethanol 95% was added (Merck, São Paulo, Brazil) for the denaturation and protein precipitation, and added 2 mL of hexane (Merck, São Paulo, Brazil) as organic solvent extraction. The aliquots were agitated for 1 minute on a vortex mixer, and centrifuged at 4,000*x* g for 10 minutes, and then the supernatant (hexane phase) obtained was transferred to another tube. This step was repeated once more, and the hexane stage evaporated on a water bath at 37ºC. For application in high performance liquid chromatography (HPLC), the dry extract was dissolved in 250 µL of dichloromethane/methanol, at a ratio of 2:1, and 20 µL were injected into the apparatus.

The concentrations of alpha-tocopherol in serum samples were determined by HPLC in gas chromatograph Shimadzu (Shimadzu Corporation, Kyoto, Japan), with pump LC 20AT Chromatograph, coupled to a detector SPD-20AT Shimadzu UV-VIS and bus *module* communicator CBM 20A Shimadzu with a LiChroCART® column, with methanol mobile phase 100%, flow 1.0 ml/min and wave length of 292 nm. The *software* for the analysis was the LC real time analysis.

The identification and quantification of alpha-tocopherol in the samples were established by comparison of the peak area obtained in the chromatogram, with the area of its pattern of alpha-tocopherol Sigma®, at a concentration of 0.29 µmol/L, and confirmed by the specific extinction coefficient (ε 1%, 1 cm =75.8 to 292 nm).^12^

The sensitivity of the method was verified by determining the detection limit, reached at a concentration of 0.03 µmol/L of alpha-tocopherol. The coefficient of variation was 0.01% at a concentration of 0.29 µmol/L for the standard and 0.05% in the concentration of 41 µmol/L for samples of milk. The accuracy of the method was evaluated by means of the recovery test, which showed recovery rate average of alpha-tocopherol of 109%. The calibration curve was performed using standard solutions of alpha-tocopherol (Sigma®). The linearity of the analytical method confirmed the determination of the statistical significance of the calibration curve coefficient in the concentration of 0.03-0.8 µmol/L (r = 0.9999).

To estimate the supply of vitamin E for the newborn, we compared the intake of milk per day with the AI of vitamin E equivalent to 4 mg/day. An intake of 396 mL for the milk and colostrum^13^, and 780 mL/day for the other phases of lactation was used, as adopted by the Institute of Medicine (acronym in Portuguese - IOM).^10^

The sample size was calculated by the software G*Power, version 3.1.9, in which the result was at least 58 participants for double-tailed test, with a significance level of 95%, power=0.8 and measure of effect=0.35.^14^

The results were presented in median (standard deviation). The normality test was applied to check the distribution of normality of the samples. To determine the difference between the averages of the different types of milk (colostrum, transitional, and mature), the analysis of variance (ANOVA) was used with the *post hoc* Tukey’s test. All differences were considered significant when p<0.05. The software used was the Statistical Package for Social Sciences (SPSS) version 13.0.

## RESULTS 

The study population was characterized by an average age of 25±5.0 years, gestational age at birth of 39.8±1.2 weeks, educational level as incomplete high school (32%), low family income, multiparous women, and who were receiving governmental social benefits (37%) (Table 1).

**Table 1:**
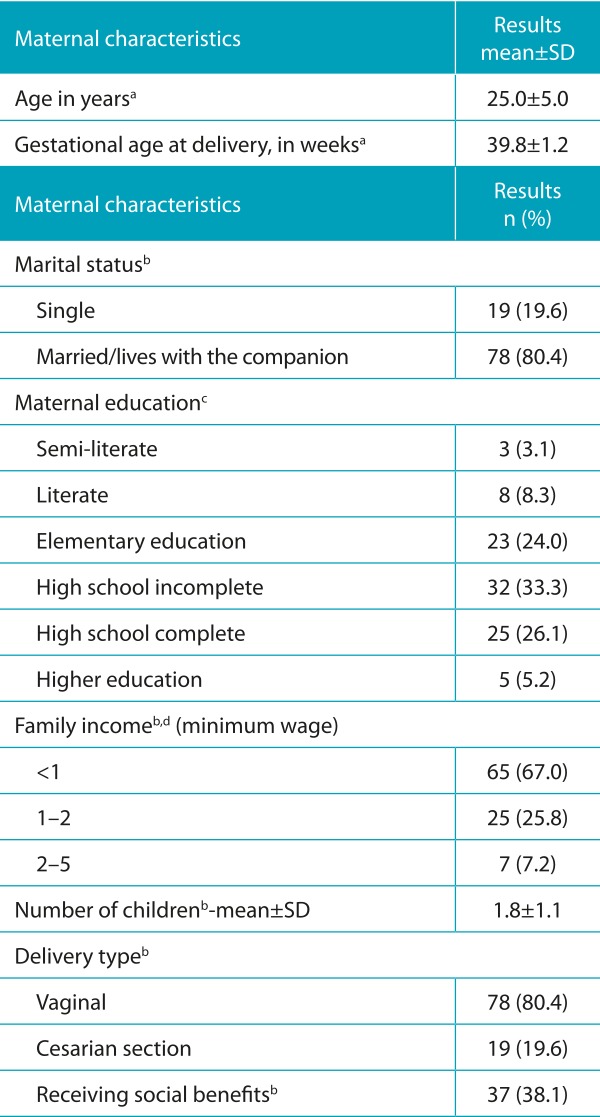
General characteristics of 100 mothers cared for childbirth in the Hospital Universitário Ana Bezerra, Santa Cruz, Rio Grande do Norte.

^a^information available for 98 cases; ^b^information available for 97 cases; ^c^information available for 96 cases; ^d^monthly income divided by the number of residents in the household. Minimum wage in Brazil equivalent to R$ 724.

**Figure 1:**
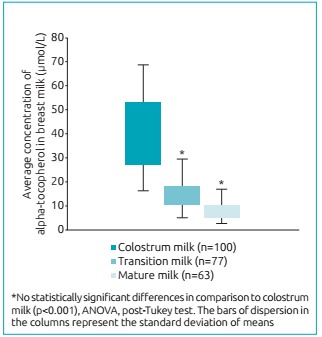
Average concentration of alpha-tocopherol in breast milk in different periods of lactation.

**Figure 2:**
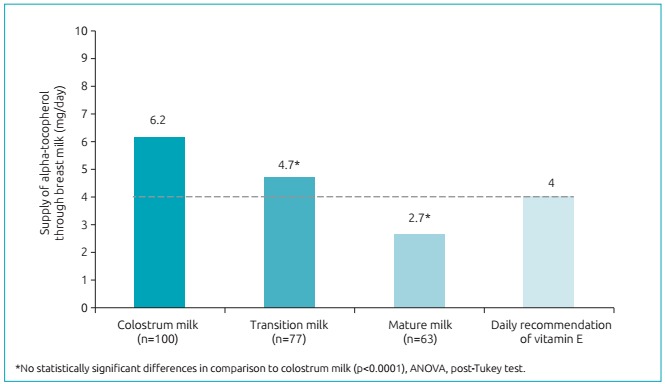
Estimated average intake of vitamin E for infants during lactation, considering the volume of milk ingested per day.

The average concentration of alpha-tocopherol decreased throughout lactation, as 40.5±15.0 µmol/L was found in colostrum milk, 13.9±5.2 µmol/L in transition milk, and 8.0±3.8 µmol/L in mature milk for 30 days after delivery (p<0.001) (Figure 1). Whereas the daily consumption potential is of 396 mL/day for colostrum, and 780 mL/day for transition and mature, the colostrum provided 6.2 mg/day, and the transition milk and mature milk, 4.7 and 2.7 mg/day, respectively (p<0.0001). This finding revealed that colostrum milk, even if a lesser volume is ingested, it provided greater amounts of vitamin E for the infant. When comparing the probable daily intake of milk with the recommendation of nutritional vitamin (4 mg/day), only the mature milk did not reach a AI proposal for the infant aged less than six months (Figure 2).

## DISCUSSION

Breastfeeding is of extreme importance for the health of the neonate, on account of its action in reducing the prevalence of infectious diseases, diarrhea, and infant mortality and the risk of allergies to milk protein, in addition to its protective role in the long term.^15^ With regard to vitamin E, breastfed children have higher levels of alpha-tocopherol reserves, which is essential during this phase of life for the development of the immune system and the lungs, the development of extracellular matrix of the vascular system, and the mental development, in addition to the prevention of hemolytic anemia and bronchopulmonary dysplasia.^16^ ^,^ ^17^ ^,^ ^18^

In general, comparing the results with the literature, it is clear that the levels of vitamin in the milk are consistent with studies involving infants in developed countries, and in situations of food safety.^7^ The concentration of alpha-tocopherol in colostrum milk was higher than that of the Polish infants,^6^ lower than that of a study conducted in Spain^9^, and similar to the concentration found in Brazil.^19^ ^,^ ^20^ With regard to the transition and mature milks, the mean concentration was similar to a longitudinal study also carried out in Brazil^8^ (Table 2).

**Table 2:**
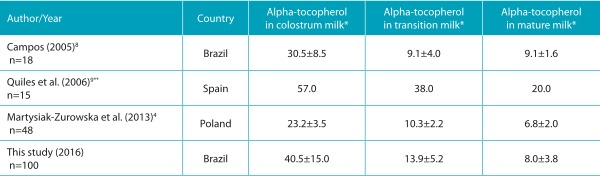
Longitudinal studies with values of alpha-tocopherol in human milk from different stages of lactation.

*Values in µmol/L, such as mean±standard deviation; **the article does not inform the value of standard deviation.

Szlagatys-Sidorkiewicz et al.^6^ suggest that higher concentrations of alpha-tocopherol in colostrum acts as a compensatory mechanism, since more of the vitamin is necessary in the first days of life according to the increased oxidative stress in the moment of birth, when the newborn comes into contact with hyperoxia, and the needs of antioxidant compounds to prevent lipid peroxidation of membranes. Another fact to add is the low body reserves of vitamin at birth because of the limited placental transfer.^1^ ^,^ ^21^

The decrease in the levels of alpha-tocopherol in milk is explained by the fact that, after the first few days of lactation, the synthesis and secretion of triglycerides increase, without a proportional increase in the secretion of phospholipids and other components of the membranes of fat globules. Thus, there is a significant reduction in the levels of alpha-tocopherol, since most of this vitamin is secreted as a constituent of the membrane of red blood cells.^2^

In the phase of mature milk, breast milk is no longer undergoing major changes in its composition, causing the levels of alpha-tocopherol to remain constant over time.^7^ Antonakou et al.^17^ showed no significant difference between the concentrations of alpha-tocopherol in mature milk among the first, third, and sixth months of lactation. Data from this study may be representative of the follow-up of lactation.

Although the amount of alpha-tocopherol offered to infants is reduced with the advancement of the phases of lactation, there is an increase in the volume of milk consumed by the newborn to meet the nutritional requirement.^7^ In this study, the mature milk has not provided the AI of vitamin E for the infant. Antonakou et al.^17^ and Kamao et al.^22^ also demonstrated that, in order to reach the stage of mature milk, the levels of alpha-tocopherol provided a lesser quantity than the nutritional recommendation of vitamin, a fact explained by the decline in the natural composition of this vitamin in milk. This study did not evaluate the possible determinants for the low concentration of alpha-tocopherol in milk, and the literature does not clearly demonstrate that the diet or the status of vitamin E feeding may influence the composition.^7^

It is worth noting that the recommendation of IOM is based on the average quantity of milk consumed by infants from 0 to 6 months (780 mL), without considering the changes in the breast milk production during the early stages of lactation.^10^ Traber^23^ says that the amount of vitamin E recommended for daily consumption is still controversial, and may be inappropriate for some population groups. In addition, most investigations about the factors that may affect the composition of alpha-tocopherol in breast milk during lactation are important to provide subsidies for the establishment of dietary intake of reference, and as a criterion in the assessment of consumption of vitamin E in infants.

The benefits of breastfeeding for the infant are indisputable, as human milk has a balanced nutritional composition, providing better bioavailability of nutrients, in addition to contain growth factors, enzymes and hormones that provide numerous immunological and psychological advantages, reduction in morbidity and mortality, among other health benefits in the long term.^24^ ^,^ ^25^ ^,^ ^26^

Considering that this study found a decrease in the concentration of alpha-tocopherol in breast milk with the advancement of the stages of lactation, and the probable lower supply of vitamin to the infant in the stage of mature milk, we suggest the development of studies that evaluate the determinant factors associated with these low levels in milk. Another important issue would be to encourage the nutritional care of the mother-child dyad in the follow-up of lactation to address a possible deficiency of this vitamin, either by encouraging the consumption of their food source, or by possible maternal supplementation with this vitamin, since there is evidence that maternal supplementation in the postpartum period may reflect an increase in the concentration of alpha-tocopherol in breast milk.^27^ There is no guidance to supplement the child with vitamin E in the wake of lactation, because their disability is poorly explored in the literature.

Finally, this study showed that vitamin E decreases in human milk with the progress of lactation, and that only the mature milk has not meet infant’s nutritional needs, according to an estimate of ingested volume of 780 mL/day. Therefore, the implementation of procedures to increase the levels of vitamin in milk would be important especially for nursing mothers living in conditions of food unsafety.
